# Linking Leaf Economic Traits With Forage Quality Across Temperate Grasslands Under Ambient and Drought Conditions

**DOI:** 10.1002/ece3.71569

**Published:** 2025-06-24

**Authors:** Taofeek O. Muraina, Amarante Vitra, Massimiliano Probo, Jason P. Martina, Alexandre Buttler, Pierre Mariotte

**Affiliations:** ^1^ Department of Biology Texas State University San Marcos Texas USA; ^2^ Department of Animal Health and Production Oyo State College of Agriculture and Technology Igbo‐Ora Oyo State Nigeria; ^3^ Ecole Polytechnique Fédérale de Lausanne (EPFL) School of Architecture, Civil and Environmental Engineering (ENAC), Laboratory of Ecological Systems (ECOS) Lausanne Switzerland; ^4^ Swiss Federal Institute for Forest Snow and Landscape Research (WSL) Lausanne Switzerland; ^5^ Department of Livestock Sciences Research Institute of Organic Agriculture (FiBL) Frick Switzerland; ^6^ Agroscope Grazing Systems Group Posieux Switzerland

**Keywords:** climate change, community‐weighted traits, herbage quality, leaf dry matter content, plant communities, specific leaf area

## Abstract

Increases in droughts may disrupt the life‐supporting services of grasslands, including the forage provision for herbivores. However, less is known about drought impacts on forage quality (i.e., dynamics of the cell characteristics of leaves and stems of herbs). Leaf economic traits reflect drought effects on plant communities, but whether they can predict forage quality patterns under drought remains unclear. We assessed the effects of early‐ and late‐season extreme droughts on (i) forage quality parameters [readily digestible, internal cellular constituents: protein, minerals, water‐soluble carbohydrate (WSC); and non‐readily digestible, cell wall components: neutral detergent fibre (NDF) and acid detergent fibre (ADF)]; (ii) community‐weighted leaf traits [specific leaf area (cwmSLA) and leaf dry matter content (cwmLDMC)]; and (iii) leaf traits–quality parameters relationships across three grasslands over two growing seasons. Both early and late droughts decreased ash and ADF and increased WSC across sites, while early drought slightly reduced protein and NDF. Both droughts decreased cwmSLA and increased cwmLDMC across sites. Community‐weighted leaf traits and forage quality parameters were unrelated under early ambient conditions, but their relationships under early‐season drought imply that lower cwmSLA and higher cwmLDMC communities had higher forage quality (higher protein and less lignified fibre contents) than higher cwmSLA and lower cwmLDMC communities. Under late‐season ambient or drought conditions, most relationships indicate that lower cwmSLA and cwmLDMC communities had higher forage quality (higher protein and ash, and more digestible fibre contents) than higher cwmSLA and cwmLDMC communities. Overall, forage quality was higher under late‐season ambient conditions compared to the early season, and both drought types had limited negative effects on forage quality in the studied grasslands. Moreover, leaf traits can predict forage quality patterns and plants' adaptation under certain circumstances, including regular intra‐seasonal dry periods and extreme drought conditions.

## Graphical abstract

We assessed the effects of early‐ and late‐season extreme droughts on (i) forage quality parameters [readily digestible, internal cellular constituents: protein, minerals, water‐soluble carbohydrate (WSC); and non‐readily digestible, cell wall components: neutral detergent fibre (NDF) and acid detergent fibre (ADF)]; (ii) community‐weighted leaf traits [specific leaf area (cwmSLA) and leaf dry matter content (cwmLDMC)]; and (iii) leaf traits–quality parameters relationships across three temperate permanent grasslands over two growing seasons. We found that forage quality was higher under late‐season ambient conditions compared to the early season, and both drought types had limited negative effects on forage quality. Moreover, leaf traits can predict forage quality patterns and plants' adaptation under certain circumstances, including regular intra‐seasonal dry periods and extreme drought conditions.

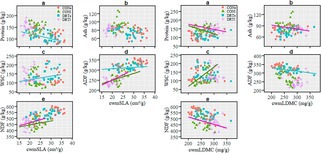

## 1 Introduction

Grasslands deliver many life‐supporting services, including provisioning of forage (i.e., leaves and stems of herbaceous plants) to herbivores (Van Coller et al. 2018; Bengtsson et al. 2019). These ecosystems depend on water for plant growth and development and are sensitive to precipitation variability (Knapp et al. 2015; Griffin‐Nolan et al. 2019). Extreme multi‐year droughts are becoming more frequent as climate change intensifies (Dai 2011; IPCC 2023), driving an increase in drought manipulation studies. Many studies have assessed drought impacts on key grassland properties, including biomass production (Cherwin and Knapp 2012; Luo et al. 2021), plant diversity (Tielbörger et al. 2014; Muraina et al. 2021) and composition (Cleland et al. 2013; Mariotte et al. 2013), plant and soil nutrients (Luo et al. 2018; Mariotte et al. 2020; Holguin et al. 2022), root biomass, and soil microbes (Holguin et al. 2022). Forage quality, defined as the nutritional composition of forage (assessed via plant cell characteristics), is another key grassland property determining the nutritional benefits herbivores derive from plant consumption (Gardarin et al. 2014; Dumont et al. 2015). However, less is known about how droughts affect forage quality, indicating the need for studies to better predict how climate change may influence herbivores' health and associated ecosystem properties.

Forage quality parameters—including protein, nutritive minerals, water soluble carbohydrate, and fibre contents (Katoch 2023)—depend on the dynamics of plant cells in leaf and stem tissues (Lemaire and Belanger 2020). In the early period of the growing season, generally high precipitation ensures high water availability that drives optimal plant cell division, expansion, and elongation, along with flexible, enlarged cell walls, leading to higher rates of photosynthesis and rapid plant growth (Feng et al. 2016). In contrast, plants gradually reduce cell elongation or attain cell maturation and increase the rigidity of cell walls towards the late season (Hamann 2012; Ezquer et al. 2020). Consequently, internal cellular contents (i.e., soluble carbohydrates, nutritive minerals, and proteins) are usually higher in early than late season, as plants increase cell wall compounds (i.e., lignin, cellulose, and hemicellulose) towards the latter period of the season (Jensen et al. 2017). Hence, forage quality is usually higher in early than late season (Lee et al. 2017; Lemaire and Belanger 2020; Perotti et al. 2021).

Drought may alter forage quality, and the extent or pattern of change may depend on the plant developmental stage and drought timing (Deléglise et al. 2015; Catunda et al. 2022). For instance, an early‐season drought may accelerate cell maturation, reducing internal cell constituents and increasing cell wall mass (Bruinenberg et al. 2002; Ren et al. 2016), preventing plants from reaching the usual high forage quality during the early season. Plants may alternatively experience slowed maturity and accumulation of non‐readily digestible cell wall components and maintain a high proportion of digestible cell contents under an early‐season drought (Buxton 1996; Deléglise et al. 2015; Catunda et al. 2022). A late‐season drought can also reduce forage quality by accelerating leaf senescence or loss and increasing the stem‐to‐leaf ratio for herbivores (Buxton 1996; Bruinenberg et al. 2002; Deléglise et al. 2015). Yet, despite recent increases in extreme droughts, the impacts of early‐ and late‐season droughts on forage quality parameters in grasslands remain unclear.

Leaves are a key forage component, and as critical organs for transpiration and carbon assimilation (Kröber et al. 2015; Tian et al. 2016), their economic traits reflect plant strategies for coping with drought conditions (Deléglise et al. 2015; Vitra et al. 2019; Blumenthal et al. 2020). The leaf economic spectrum describes an investment strategy where multiple traits [such as specific leaf area (SLA) and leaf dry matter content (LDMC)] co‐vary to maximise plant fitness using one of the two main strategies: conservative resource‐use and slow growth versus acquisitive resource‐use and fast return on investment (Wright et al. 2004). Under drought conditions, some plants can conserve water by reducing leaf area (Wellstein et al. 2017) for reduced transpiration and photosynthesis (Nord and Lynch 2009). Some plants can increase water absorption and translocation from belowground to aboveground organs (Lombardini and Rossi 2019), expanding leaf area for enhanced photosynthesis and allocating resources belowground for sustainable growth under drought (Wellstein et al. 2017; Blumenthal et al. 2020). Plants may also invest more in structural leaf components (often measured as LDMC) to increase thickness and compactness under drought, reducing transpiration and photosynthesis (Poorter et al. 2009; Wellstein et al. 2017). These strategies indicate that SLA and LDMC mediate plant photosynthetic capacity, growth rate (Reich et al. 1997; Hulshof et al. 2013; Firn et al. 2019), water‐use strategy (Wright et al. 2001; Wellstein et al. 2017), and other grassland functions and properties, including forage quality. Yet, empirical evidence linking leaf economic traits with forage quality parameters under extreme drought conditions is limited, making the investigation of these relationships critical for a better understanding of drought effects on grasslands.

Here, we simulated early‐ and late‐season droughts in three permanent grasslands in the Swiss Jura Mountains over two consecutive growing seasons. Early‐season drought involved a 70% reduction in ambient precipitation during the first two‐month growth cycle of a six‐month growing season, while late‐season drought reduced the same precipitation during the second two‐month growth cycle. We addressed three questions: (1) how do forage quality parameters—internal cellular constituents (proteins, minerals, water soluble carbohydrate) and cell wall components (neutral detergent fibre and acid detergent fibre)—and leaf economic traits [community‐weighted (cwm) SLA and LDMC] change from early to late periods of the season under ambient conditions; (2) how do forage quality parameters and leaf traits respond to early‐ and late‐season droughts; and (3) how do community‐weighted leaf traits and forage quality parameters relate under early‐ and late‐season ambient and drought conditions. We hypothesised that (1) under ambient conditions, forage quality would decrease (i.e., decreased cellular contents, increased cell wall contents) from early to late season (Lee et al. 2017; Lemaire and Belanger 2020; Perotti et al. 2021), with lower cwmSLA and higher cwmLDMC in late season (Vitra et al. 2019); (2) both early‐ and late‐season droughts would greatly decrease forage quality by decreasing plants' water acquisitive and use capacity (decreased cwmSLA, increased cwmLDMC; Luo et al. 2023; Song et al. 2024), with variations among sites due to differing species composition and drought tolerance (Song et al. 2022, 2024); and (3) under both early‐ and late‐season ambient conditions, cwmSLA would positively correlate with internal cellular constituents and negatively with cell wall components, while cwmLDMC would show negative and positive relationships with the internal cellular and cell wall parameters, respectively. These relationships are expected to change under both drought conditions as plants exhibit their adaptation strategies.

## 2 Material and Methods

### 2.1 Study Sites

This study was conducted in three permanent grasslands across the Jura Mountains in Switzerland over two consecutive growing seasons (spring 2015 to fall 2016). The grasslands were located at Chéserex (site A; N 46°24′, E 6°10′), Saint‐George (site B; N 46°30′, E 6°15′), and Trois Chalets (site C; N 46°53′, E 6°21′). The sites were selected along an altitudinal gradient, from 540 m a.s.l. (site A) to 945 m (site B) to 1330 m (site C). The long‐term (1984–2013) range of mean annual precipitation (MAP) includes 647–1398 mm (site A), 1226–1442 mm (site B), and 1206–2453 mm (site C), while the mean annual temperature (MAT) range includes 8.2°C–12.0°C (site A), 6.6°C–8.8°C (site B), and 5.3°C–7.8°C (site C) (MeteoSuisse, Switzerland). The long‐term MAP and MAT followed a gradient, ranging from 1050 mm and 10.4°C (site A) to 1290 mm and 7.6°C (site B) to 1952 mm and 6.5°C (site C). Hence, sites A, B, and C represent the driest‐hottest, medium wet‐hot, and wettest‐coolest grasslands, respectively.

The soil type at the three sites was classified as Cambisols (World Reference Base for Soil Resources—IUSS Working Group WRB, 2006). The dominant plant species at site A—accounting for about 68% of total plant cover under ambient conditions over the two years (Table S2)—included four perennial grasses ( *Lolium perenne* L., *Dactylis glomerata* L., *Poa pratensis* L., *Phleum pratense* L.), one legume ( *Trifolium repens* L.), and one non‐legume forb ( *Taraxacum officinale* aggr.). All the species that dominated site A, except *Phleum pratense* and *Taraxacum officinale*, also dominated site B and accounted for about 66% of total plant cover. The dominant species at site C were two grasses ( *Agrostis capillaris* L., *Festuca rubra* aggr.), one legume ( *Trifolium repens* L.), and two non‐legume forbs ( *Ranunculus acris* L., *Alchemilla vulgaris* aggr.) and they accounted for about 81% of total plant cover (Table S2). Additional information about the three sites can be found in previous studies (Buttler et al. 2019; Vitra et al. 2019).

### 2.2 Study Design and Drought Simulation

In each site, we established three drought treatments (i.e., precipitation manipulation) during the two growing seasons. The treatments included control (no drought), early‐season drought, and late‐season drought. In each site, five 12 m × 6 m grassland blocks were established, and each block included three similar plots representing the three treatments. Each plot had a size of 4 m × 0.9 m and two neighboring plots had at least 80 cm spacing. Throughout the growing season, each block (including control and drought plots) was completely covered with a rainout shelter. Thus, we simulated all the treatments under each shelter by manually adding varied quantities of water to the plots. Each rainout shelter had a transparent plastic film roof (180 μm, transparent M42, Filclair, Numeris 6.40, Venelles, France), 12 m length, 6.4 m width, and 3 m height. The rainout shelters had a minimal impact on the light environment, allowing over 90% of photosynthetically active radiation to reach the plants.

To simulate the treatments each year, water was manually supplied to each plot using a 0.9 m‐long, metered sprinkler bar (matching the plot width), which was connected via a hose to the water network (site A and B) or to a portable water tank (site C). Water was added to the control plots based on the pre‐2015 thirty‐year MAP at each site (MeteoSuisse, Switzerland). During the first 2 months of the growing season, spanning the onset and peak of plant biomass production under ambient conditions, control plots received 143, 234, and 240 mm of precipitation at sites A, B, and C, respectively. During the following two months of the season (i.e., after the peak of biomass production), control plots received 154, 213, and 272 mm at sites A, B, and C, respectively. Dates of the year, mean daily air temperature, deviation from the long‐term average, and vapour pressure deficit during the different growth periods at the three sites and for both years are available in Table S1. Control plots were watered at 2–3 days intervals, in line with the long‐term rainfall frequency of the region, corresponding to 11 rainy days per month (CH 2018). Unlike the control plots, early‐ and late‐drought plots received 30% of the water applied to the control plots during the first and second 2‐month growth cycles, respectively. During the drought months, the drought plots were watered at 4–5 days intervals to achieve about a 50% decrease in rainfall frequency, which is expected together with the decreased precipitation (CH 2018). During the non‐drought months, the early‐ and late‐drought plots received the same amount of water in the same day intervals as control plots.

### 2.3 Experimental Field Management

The experimental fields at the three sites were managed similarly. Organic manure (with 5.2% organic nitrogen and 4.4% phosphate) was applied to each plot at a rate of 150 kg N ha^−1^ and 125 kg P ha^−1^ in split applications, half in March and half in October of each experimental year according to the Swiss recommendations for permanent grasslands managed for hay production (Sinaj et al. 2009). Because mowing every two months is a common practice to promote higher yield in the studied medium‐intensity managed grasslands, all plots were mowed thrice at two‐month intervals to a height of 5 cm in each experimental year. The first and second mowing coincided with the end of early‐ and late‐drought periods, respectively. A cleaning cut was performed before the winter season of the first year—i.e., two months after the second harvest—after which the rainout shelters were removed.

### 2.4 Plant Biomass Sampling and Forage Quality Analysis

To assess the impacts of early and late droughts on forage quality, we clipped aboveground plant biomass at 5 cm above ground level within a 65 × 400 cm subplot in each plot at the end of the first and second 2‐month growth cycles in each year. The biomass samples were oven‐dried at 60°C for 72 h, ground to pass a 1‐mm screen (Brabender rotary mill; Brabender GmbH & Co. KG, Duisburg, Germany), and analyzed in the laboratory for forage quality parameters. Using the Van Soest method (Van Soest et al. 1991), NDF (ISO 16472:2006) and ADF (ISO 13906:2008) were determined gravimetrically (ISO 6865:2000) after alkaline and acid digestions of the samples in a fibre analyser (Fibretherm Gerhardt FT‐12, C. Gerhardt GmbH & Co. KG, Königswinter, Germany). We evaluated the nitrogen (N) content using the Dumas method (ISO 16634‐1:2008; Jimenez and Ladha 1993) and calculated the crude protein (simply referred to as protein in this paper) as *N* × 6.25. Ash content was determined after incineration at 550°C until a stable mass was reached according to ISO 5984_2002 (prepASH, Precisa Gravimetrics AG, Dietikon, Switzerland). WSC was determined by spectrophotometry after a colourimetric reaction (Hall et al. 1999).

### 2.5 Plant Community Composition

A few days before the first and second biomass sampling at the end of the early and late drought periods, respectively, plant species composition of each plot was surveyed using the Daget‐Poissonet method (Daget and Poissonet 1971). Four 400 cm transects spaced 20 cm apart were set up within each experimental plot, leaving a 10 cm border to avoid edge effects. Along each transect, twenty census points were established at regular intervals, yielding 80 points per plot in total. We then placed a 1 mm dagger on each census point and recorded the names of all plant species in contact with the edge of the dagger. The relative species abundance was thereafter estimated by dividing the number of occurrences of each species in each plot by the total number of occurrences of all species in that plot.

### 2.6 Community‐Weighted Leaf Economic Traits

A few days before biomass sampling, we collected leaves of each of the dominant species in each site (*see* dominant species identity in the ‘study site’ section above) and each plot. We then measured both LDMC and SLA according to the protocol of Cornelissen et al. (2003), as described in a previous study (Vitra et al. 2019). In each experimental plot at each site, the youngest and healthy fully mature leaf on five randomly selected mature individuals was sampled for each dominant species (i.e., 5 leaves per dominant species per plot). The leaves were kept in plastic bags containing a piece of paper towel moisturised with deionised water for 24 h at 4°C to rehydrate the leaf tissues (Garnier et al. 2001). At the end of rehydration time, each leaf was weighed to determine water‐saturated fresh weight (FW), and then dried at 60°C for 72 h to determine dry weight (DW). Following the determination of FW and DW, LDMC (mg g^−1^) of each leaf was calculated as DW (mg) divided by FW (g), while the dried leaves were also used for SLA determination. We first placed each dried leaf in a planimeter (LI‐COR, LI 3000C Portable Area Meter) to measure the leaf surface area. Thereafter, we calculated SLA (cm^2^ g^−1^) for each leaf as the leaf surface area (cm^2^) divided by DW (g).

We calculated the community‐weighted mean (cwm) of LDMC and SLA for each plot (Garnier et al. 2001) in three steps. We first calculated the mean of the five values of LDMC or SLA recorded for each dominant species in each plot. Second, we multiplied LDMC or SLA values (${\text{trait}}_{i}$) by the relative abundance value ($p_{i}$) of the corresponding species in that plot. As shown below, we finally calculated the cwmLDMC or cwmSLA for each experimental plot as the sum of all the values obtained from the species‐level multiplication of LDMC or SLA value by relative abundance value (in step two) divided by the sum of the relative abundances of all the selected dominant species in that plot.

$${\mathrm{cwm}}_{\text{trait}}=\frac{\;\sum_{i=1}^{n}p_{i}\times {\text{trait}}_{i}}{\sum p_{i}}$$

where $p_{i}$ is the relative abundance of each selected dominant species i in a plot, n the number of dominant species, and ${\text{trait}}_{i}$ the value of a given trait for a dominant species i.

### 2.7 Statistical Analyses

All analyses were performed in R studio (version 4.2.1; R Core Team 2022). Our analyses were conducted in three stages in line with the three questions outlined in the introduction. First, we used control plots data to test how forage quality parameters and leaf traits differ between early‐ and late‐season ambient conditions. To test this intra‐season time effect on each quality parameter or trait across the sites, we used mixed‐effects ANOVA models with *lme* function (Pinheiro et al. 2018), with ‘time’ and ‘site’ as interactive fixed effects, ‘year nested in block’ as a random effect (Table 1). For the within site tests, each model had ‘time’ as a fixed effect, and ‘year nested in block’ as a random effect (Table 1).

**TABLE 1:** Results of analysis of variance (ANOVA) for effects of growing season time (early vs. late) on forage quality parameters and community‐weighted leaf economic traits across and within three mountain grasslands over 2 years.

Variables	DF	Protein (g/kg)		Ash (g/kg)		WSC (g/kg)		ADF (g/kg)		NDF (g/kg)		cwmSLA (cm^2^/g)		cwmLDMC (mg/g)	
		*F*	*p*	*F*	*p*	*F*	*p*	*F*	*p*	*F*	*p*	*F*	*p*	*F*	*p*
Time	1,43	61.47	**<.0001**	26.05	**< .0001**	2.88	*0.0971*	112.45	**< .0001**	101.03	**<.0001**	77.20	**< .0001**	2.65	0.111
Site	2,43	11.95	**0.0001**	28.19	**<.0001**	4.11	**0.0232**	26.29	**<.0001**	27.75	**<.0001**	34.67	**<.0001**	34.09	**<.0001**
Time × Site	2,43	1.04	0.361	0.004	0.996	10.87	**0.0002**	1.53	0.2285	0.32	0.726	10.21	**0.0002**	4.11	**0.023**
Site A	1,7	24.82	**0.0016**	30.40	**0.0009**	1.47	0.2649	30.69	**0.0009**	33.09	**0.0007**	34.66	**0.0006**	4.09	*0.083*
Site B	1,9	19.86	**0.0016**	32.59	**0.0003**	15.78	**0.0032**	34.99	**0.0002**	89.11	**<.0001**	193.69	**<.0001**	5.59	**0.042**
Site C	1,9	24.82	**0.0001**	6.94	**0.0271**	6.47	**0.0316**	60.11	**<.0001**	65.11	**<.0001**	2.97	0.119	1.99	0.192

*Note:* This table presents the analyses for ambient (control) treatment in Figures 1, 2 and S1–S4. Significant (*p* < 0.05) and marginally significant (*p* < 0.1) *p*‐values are shown in bold and italics, respectively. Site A: Chéserex, Site B: Saint‐George, and Site C: Trois Chalets.

Abbreviations: ADF, acid detergent fibre; cwmLDMC, community‐weighted leaf dry matter content; cwmSLA, community‐weighted specific leaf area; DF, degree of freedom; *F*, *F*‐value; NDF, neutral detergent fibre; WSC, water soluble carbohydrate.

Second, we tested the effects of early‐ and late‐season droughts on forage quality parameters and leaf traits across and within sites. Given that forage quality and leaf traits often change between early and late season periods under ambient conditions due to plant maturation (as our first analysis corroborated), we separately assessed the effects of early and late droughts across and within the sites. To test the early or late drought effect across sites, we used mixed‐effects ANOVA models with the *lme* function, drought ‘treatment’ and ‘site’ as interactive fixed effects, and ‘year nested in block’ as a random effect (Table 2). Each *lme* model for the within‐site early or late drought effect included drought ‘treatment’ as a fixed effect and ‘year nested in block’ as a random effect (Table 2).

**TABLE 2:** Results of analysis of variance (ANOVA) for drought effects at different growing season times on forage quality parameters and community‐weighted leaf economic traits across and within three mountain grasslands over 2 years.

Variables	DF	Protein (g/kg)		Ash (g/kg)		WSC (g/kg)		ADF (g/kg)		NDF (g/kg)		cwmSLA (cm^2^/g)		cwmLDMC (mg/g)	
		*F*	*p*	*F*	*p*	*F*	*p*	*F*	*p*	*F*	*p*	*F*	*p*	*F*	*p*
Early season															
Across sites:															
Drought (D)	1,42	3.34	*0.075*	4.04	*0.051*	11.71	**0.001**	9.19	**0.004**	4.56	**0.039**	10.92	**0.002**	6.32	**0.016**
Site (S)	2,42	3.92	**<.0001**	6.71	**<.0001**	16.51	**< .0001**	13.42	**<.0001**	23.70	**<.0001**	84.00	**<.0001**	44.82	**< .0001**
D × S	2,42	1.22	0.305	1.15	0.327	0.88	0.421	3.17	*0.052*	4.74	**0.014**	0.64	0.533	1.00	0.376
Site‐level:															
Site A	1,7	0.19	0.675	0.03	0.858	0.99	0.352	6.74	**0.036**	7.543	**0.029**	1.48	**0.012**	0.57	0.476
Site B	1,9	1.21	0.300	4.08	*0.074*	95.08	**<.0001**	14.34	**0.004**	7.86	**0.021**	48.59	**0.015**	48.59	**0.0001**
Site C	1.9	5.13	**0.049**	12.99	**0.006**	15.78	**0.003**	0.12	0.738	4.34	*0.067*	1.49	0.252	1.59	0.238
Late season															
Across sites:															
Drought (D)	1,45	0.54	0.467	11.81	**0.001**	6.82	**0.012**	31.45	**<.0001**	0.01	0.946	25.37	**<.0001**	42.36	**< .0001**
Site (S)	2,45	16.81	**< .0001**	23.77	**< .0001**	16.57	**<.0001**	26.84	**<.0001**	20.15	**<.0001**	2.74	*0.076*	8.94	**0.0005**
D × S	2,45	3.67	**0.033**	2.64	*0.082*	0.36	0.701	17.49	**<.0001**	0.25	0.784	2.10	0.134	2.08	0.136
Site‐level:															
Site A	1,9	0.05	0.823	2.04	0.187	10.74	**0.009**	3.99	*0.077*	0.01	0.926	4.95	*0.053*	12.94	**0.006**
Site B	1,9	24.53	**0.0008**	15.38	**0.004**	93.15	**<.0001**	124.06	**<.0001**	0.74	0.413	31.17	**0.0003**	52.36	**<.0001**
Site C	1,9	7.49	**0.023**	9.91	**0.012**	12.94	**0.006**	0.30	0.597	0.36	0.562	21.04	**0.001**	17.59	**0.002**

*Note:* This table presents the analyses for both ambient (control) and drought treatments in Figures 1, 2 and S1–S4. Significant (*p* < 0.05) and marginally significant (*p* < 0.1) *p*‐values are shown in bold and italics, respectively. Site A: Chéserex, Site B: Saint‐George, and Site C: Trois Chalets.

Abbreviations: ADF, acid detergent fibre; cwmLDMC, community‐weighted leaf dry matter content; cwmSLA, community‐weighted specific leaf area; DF, degree of freedom; *F*, *F*‐value; NDF, neutral detergent fibre; WSC, water soluble carbohydrate.

Finally, we used linear mixed‐effect regression models with *lme* function to assess the relationships between forage quality parameters and leaf traits under ambient and drought conditions in early and late periods of the season. Each relationship was assessed through a *lme* model with a quality parameter as response variable, a leaf trait as explanatory variable, and ‘year nested in block and site’ as a random effect (Tables 3 and 4). Prior to each aforementioned analysis, we conducted Shapiro–Wilk test of normality for all response variables' data and log‐transformed those that failed the test to improve normality. However, given that the results of the transformed and untransformed data were qualitatively similar, the results obtained from the analyses of the original data are reported for uniformity and better interpretation.

**TABLE 3:** Relationships between community‐weighted specific leaf area (cwmSLA) and forage quality parameters across three mountain grasslands at different growing season times over 2 years.

Relationship	Treatment	Early season					Late season				
		Regression eqn.	DF	*R* ^2^m	*R* ^2^c	*p*	Regression eqn.	DF	*R* ^2^m	*R* ^2^c	*p*
cwmSLA‐Protein	Control	*y* = 155.39 − 1.39*x*	12	0.10	0.99	0.108	*y* = 133.29 + 0.65*x*	14	0.01	0.99	0.550
	Drought	*y* = 159.30 − 1.80*x*	13	0.15	0.99	**0.046**	*y* = 145.92 + 0.26*x*	14	0.00	0.99	0.804
cwmSLA‐Ash	Control	*y* = 69.05 + 0.28*x*	12	0.01	0.99	0.633	*y* = 77.80 + 0.53*x*	14	0.01	0.99	0.493
	Drought	*y* = 72.51 + 0.08*x*	13	0.00	0.99	0.894	*y* = 75.70 + 0.37*x*	14	0.01	0.96	0.219
cwmSLA‐WSC	Control	*y* = 90.67 + 1.21*x*	12	0.03	0.99	0.381	*y* = 191.61 − 3.22*x*	14	0.05	0.99	0.224
	Drought	*y* = 64.71 + 2.89*x*	13	0.12	0.99	*0.073*	*y* = 167.69 − 1.68*x*	14	0.01	0.99	0.494
cwmSLA‐ADF	Control	*y* = 336.92 − 0.19*x*	12	0.00	0.99	0.862	*y* = 162.81 + 4.77*x*	14	0.17	0.99	**0.013**
	Drought	*y* = 285.61 + 1.21*x*	13	0.11	0.99	*0.097*	*y* = 150.44 + 5.06*x*	14	0.19	0.94	**0.006**
cwmSLA‐NDF	Control	*y* = 551.77 − 0.11*x*	12	0.00	0.96	0.953	*y* = 366.73 + 4.36*x*	14	0.07	0.94	*0.084*
	Drought	*y* = 476.36 + 2.12*x*	13	0.09	0.99	0.141	*y* = 367.57 + 4.92*x*	14	0.08	0.94	**0.026**

*Note:* This table presents the fixed effects results for Figure 3a–e. Significant (*p* < 0.05) and marginally significant (*p* < 0.1) *p*‐values are shown in bold and italics, respectively. *R* ^2^m and *R* ^2^c‐ Marginal (m) and conditional (c) coefficient of determination (R^2^) indicate the variance explained by fixed effect alone, and that explained by both fixed and random effects, respectively.

Abbreviations: ADF, acid detergent fibre; DF, degree of freedom; NDF, neutral detergent fibre; WSC, water soluble carbohydrate.

**TABLE 4:** Relationships between community‐weighted leaf dry matter content (cwmLDMC) and forage quality parameters across three mountain grasslands at different growing season times over 2 years.

Relationship	Treatment	Early season					Late season				
		Regression eqn.	DF	*R* ^2^m	*R* ^2^c	*p*	Regression eqn.	DF	*R* ^2^m	*R* ^2^c	*p*
cwmLDMC‐Protein	Control	*y* = 90.24 + 0.09*x*	12	0.03	0.99	0.478	*y* = 235.40 − 0.34*x*	14	0.09	0.99	**0.034**
	Drought	*y* = 104.67 + 0.02*x*	13	0.00	0.99	0.889	*y* = 230.60 − 0.27*x*	14	0.09	0.99	**0.032**
cwmLDMC‐Ash	Control	*y* = 80.79 − 0.01*x*	12	0.00	0.99	0.834	*y* = 118.47 − 0.11*x*	14	0.02	0.99	0.338
	Drought	*y* = 90.43 − 0.06*x*	13	0.03	0.99	0.333	*y* = 111.47 − 0.10*x*	14	0.06	0.97	**0.019**
cwmLDMC‐WSC	Control	*y* = 163.57 − 0.15*x*	12	0.02	0.99	0.422	*y* = −93.65 + 0.81*x*	14	0.21	0.99	**0.022**
	Drought	*y* = 182.43 − 0.15*x*	13	0.02	0.99	0.556	*y* = 96.06 + 0.13*x*	14	0.01	0.99	0.663
cwmLDMC‐ADF	Control	*y* = 345.32 − 0.06*x*	12	0.01	0.99	0.654	*y* = 364.31 − 0.35*x*	14	0.06	0.99	0.222
	Drought	*y* = 368.87 − 0.19*x*	13	0.15	0.99	**0.045**	*y* = 237.97 + 0.05*x*	14	0.00	0.99	0.834
cwmLDMC‐NDF	Control	*y* = 519.13 + 0.12*x*	12	0.01	0.97	0.609	*y* = 503.02 – 0.14*x*	14	0.00	0.94	0.696
	Drought	*y* = 595.73 − 0.23*x*	13	0.07	0.99	0.186	*y* = 620.43 – 0.53*x*	14	0.07	0.94	*0.080*

*Note:* This table presents the fixed effects results for Figure 4a–e. Significant (*p* < 0.05) and marginally significant (*p* < 0.1) *p*‐values are shown in bold and italics, respectively. *R* ^2^m and *R* ^2^c‐ Marginal (m) and conditional (c) coefficient of determination (*R* ^2^) indicate the variance explained by fixed effect alone, and that explained by both fixed and random effects, respectively.

Abbreviations: ADF, acid detergent fibre; DF, degree of freedom; NDF, neutral detergent fibre; WSC, water soluble carbohydrate.

## 3 Results

### 3.1 Effects of Time of the Season on Forage Quality

**FIGURE 1:**
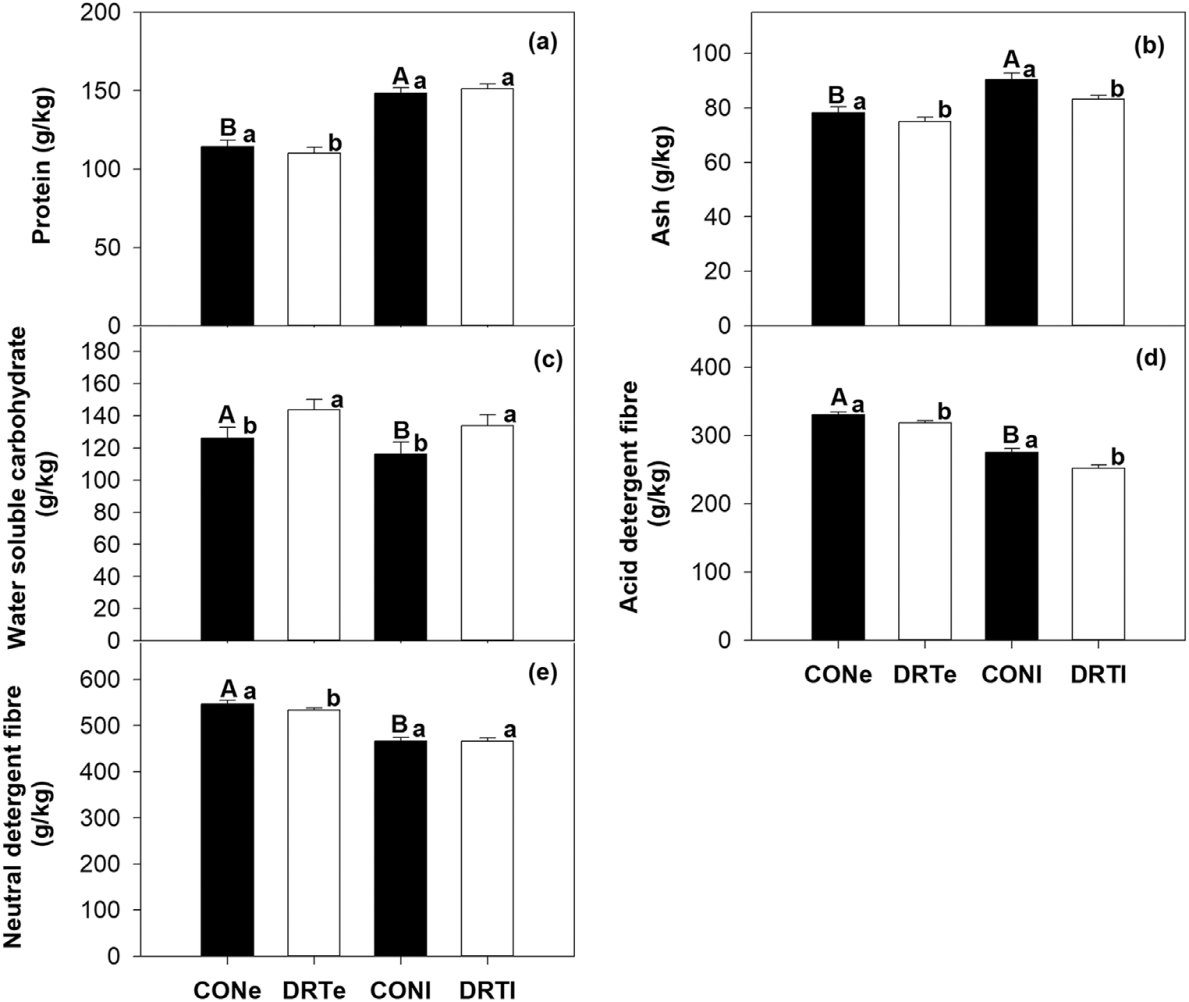
Seasonal drought effects (early season and late season) on forage quality parameters across three mountain grasslands. Average values over 2 years and SE are given. CONe is ambient (control) conditions at early season period (i.e., before peak of biomass production); CONl is ambient (control) conditions at late season period (i.e., after peak of biomass production); DRTe is drought treatment conditions at early season period and DRTl is drought treatment conditions at late season period. Different upper‐case letters on black bars indicate significant (*p* < 0.05) or marginal (*p* < 0.1) differences between early and late season periods under ambient conditions in control plots (Table 1). Different lower‐case letters on black versus white bar indicate significant differences between control and drought treatments at either early or late period of the season (Table 2).

Under ambient conditions, forage quality parameters (protein, ash, WSC, ADF, and NDF contents) changed significantly (*p* < 0.05) or marginally (WSC: *p* < 0.1) with the time (T) of the season (early vs. late) across sites (Table 1; Figure 1a–e). The time effect on each parameter, except WSC, did not vary between sites (S) (non‐significant T × S interactions for other than WSC; Table 1; Figure S1). Across sites, protein and ash contents increased by ~14% and 13%, respectively, from early to late season (Figure 1a,b; Table 1). In contrast, WSC, ADF, and NDF decreased by 8%, ~17%, and 15%, respectively, from early to late season across sites (Figure 1c–e; Table 1).

Protein significantly increased by ~20%, ~39%, and 29% from early to late season at the driest‐hottest site (A), the medium wet‐hot site (B), and the wettest‐coolest site (C), respectively (Figure S1a–c; Table 1). Similarly, ash increased by ~32%, ~16%, and ~15% at sites A, B, and C, respectively (Figure S1d–f; Table 1). WSC was similar between seasons at site A, decreased by ~35% in late season at site B, and increased by ~25% in late season at site C (Figure S2a–c; Table 1). ADF was 17%, ~13%, and ~21% lower in late season at sites A, B, and C, respectively (Figure S3a–c; Table 1). Similarly, NDF was ~14%, ~16%, and ~15% lower in late season at sites A, B, and C, respectively (Figure S3d–f; Table 1).

### 3.2 Effects of Time of the Season on Leaf Traits

**FIGURE 2:**
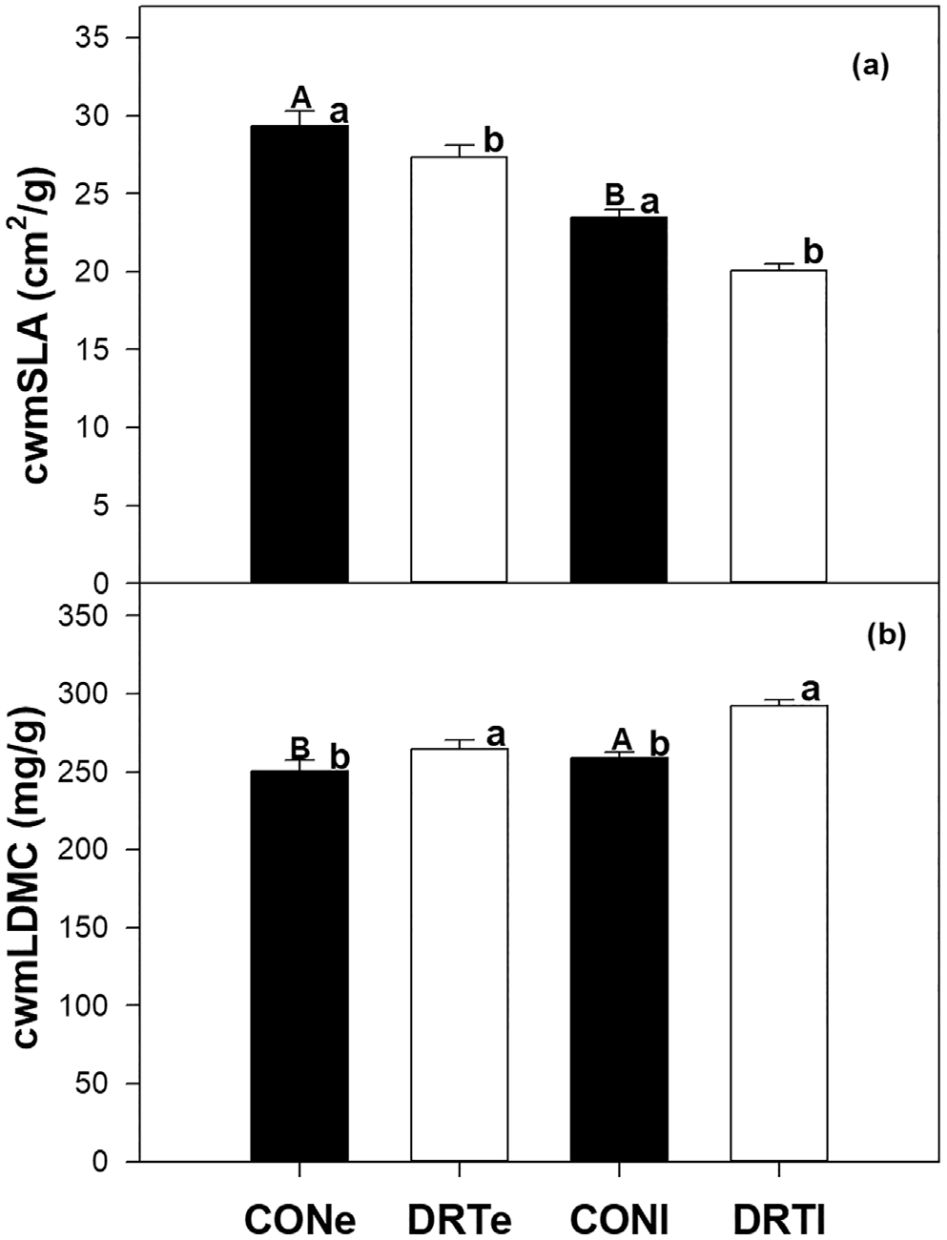
Seasonal drought effects (early season and late season) on community‐weighted leaf traits (cwmSLA and cwmLDMC) across three mountain grasslands. Average values over 2 years and SE are given. CONe is ambient (control) conditions at early season period (i.e., before peak of biomass production); CONl is ambient (control) conditions at late season period (i.e., after peak of biomass production); DRTe is drought treatment conditions at early season period; DRTl is drought treatment conditions at late season period. Different upper‐case letters on black bars indicate significant (*p* < 0.05) or marginal (*p* < 0.1) differences between early and late season periods under ambient conditions (Table 1). Different lower‐case letters on black versus white bar indicate significant differences between control and drought treatments at early or late period of the season (Table 2).

Time of the season significantly affected cwmSLA under ambient conditions (Figure 2a) and this effect varied between sites (Table 1). Across sites, cwmSLA decreased by 20% from early to late season (Figure 2a; Table 1). Within sites, cwmSLA was similar between seasons at site C but was 26% and 24% lower in late season at sites A and B, respectively (Figure S4a–c; Table 1). However, cwmLDMC increased by 3% from early to late season across the sites (Figure 2b), and this effect varied among sites (significant T × S interaction; Table 1). At site C, cwmLDMC remained similar between seasons, but it was ~10% and ~8% higher in late season at sites A and B, respectively (Figure S4d–f; Table 1).

### 3.3 Effects of Early and Late Droughts on Forage Quality

Early drought (D) significantly or marginally impacted all forage quality parameters across sites, with both fibre variables showing significant drought × site interactive effects (Table 2). In contrast, late drought significantly changed ash, WSC, and ADF across sites, but did not impact protein and NDF (Table 2). Moreover, the late drought effect on protein, ash, and ADF varied with site (significant D × S interactions; Table 2).

Across sites, early drought marginally decreased protein content by ~4%, while late drought did not alter it (Figure 1a; Table 2). Early and late droughts marginally and significantly reduced ash content across sites by 4% and ~8%, respectively (Figure 1b; Table 2). In contrast, early and late droughts significantly increased WSC content across sites by ~14% and 15%, respectively (Figure 1c; Table 2). Like ash, early and late droughts significantly decreased ADF across sites by ~4% and ~9%, respectively (Figure 1d; Table 2). NDF content significantly changed only under early drought, with a 2% decrease (Figure 1e; Table 2).

Within sites, early drought affected protein only at site C, significantly decreasing it by 4% (Figure S1a–c; Table 2). Late drought significantly increased protein by ~11% at site B, but decreased it by 5% at site C. Early drought marginally decreased ash at site B (~5% decline) and significantly at site C (~6%), while late drought significantly decreased ash by ~5% and ~14% at sites B and C, respectively (Figure S1d–f; Table 2). Early drought significantly increased WSC at sites B (~19% increase) and C (~10%), while late drought increased it at sites A (~10%), B (~30%), and C (~11%) (Figure S2a–c; Table 2). Early drought marginally and significantly decreased ADF at sites A (~5% decline) and B (6%), respectively, while late drought significantly decreased it at sites A (3%) and B (19%) (Figure S3a–c; Table 2). Only early drought impacted NDF, significantly decreasing it by 5% at sites A and B and marginally increasing it by 2% at site C (Figure S3d–f; Table 2).

### 3.4 Effects of Early and Late Droughts on Leaf Traits

Early drought significantly affected leaf traits across sites and the effects did not depend on site (non‐significant treatment × site interaction; Table 2). Across sites, early and late droughts decreased cwmSLA by 7% and 15%, respectively (Figure 2a; Table 2), but increased cwmLDMC by 5% and 11%, respectively (Figure 2b; Table 2). Early drought decreased cwmSLA at sites A (9% decline) and B (~7%) (Figure S4a–c; Table 2), but late drought decreased it at sites A (−11%), B (−21%), and C (−11%) (Figure S4a–c; Table 2). Early drought increased cwmLDMC at site B (12%) (Figure S4a–c; Table 2) while late drought increased it at site A (10%), site B (20%), and site C (9%) (Figure S4a–c; Table 2).

### 3.5 Relationships Between Leaf Traits and Forage Quality With and Without Droughts

**FIGURE 3:**
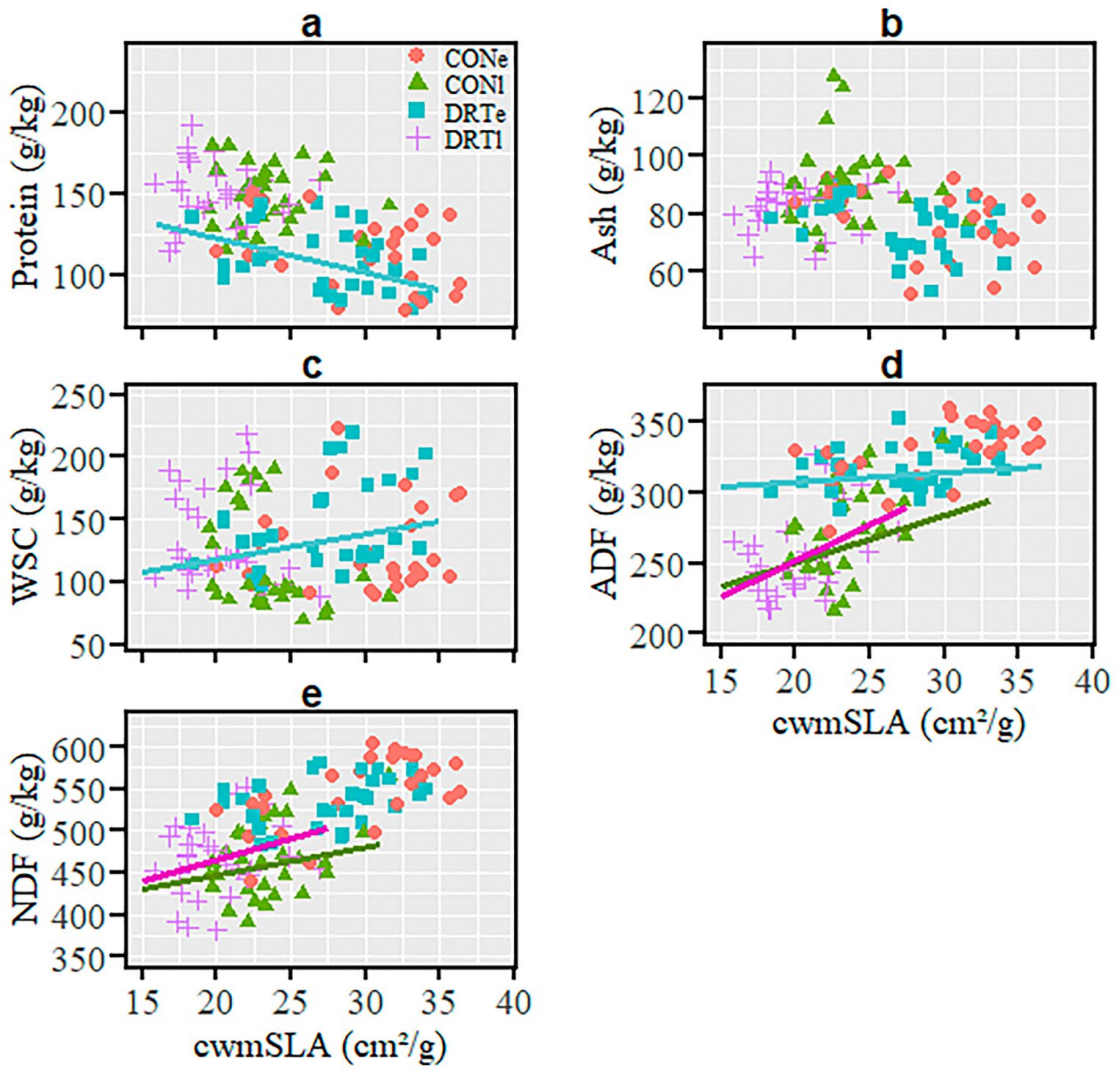
Relationships between community‐weighted mean specific leaf area (cwmSLA) and forage quality parameters across three grasslands at different times of the season (early and late) over 2 years. Only relationships (regression lines) with significant (*p* < 0.05) or marginal (*p* < 0.1) *p*‐values are shown in the panels a–e while the full results are presented in Table 3. Each colour of the data points and regression lines represents each treatment, as indicated in the legends. CONe is ambient (control) conditions at the early season period (i.e., before peak of biomass production); CONl is ambient (control) conditions at the late season period (i.e., after peak of biomass production); DRTe is drought treatment conditions at the early season period and DRTl is drought treatment conditions at the late season period. ADF, acid detergent fibre; NDF, neutral detergent fibre; WSC, water soluble carbohydrate.

In early season, protein content did not relate to cwmSLA under ambient conditions; however, it negatively related to cwmSLA under drought conditions (Figure 3a; Table 3). Ash and NDF contents were unrelated to cwmSLA under both early‐season ambient and drought conditions (Figure 3b,e; Table 3). In early season, cwmSLA was not related to WSC or ADF under ambient conditions but had marginal positive relationships with both under drought (Figure 3c,d; Table 3). In late season, protein, ash, and WSC contents did not relate to cwmSLA under ambient and drought conditions (Figure 3a–c; Table 3); however, positive cwmSLA–ADF relationships were found under both conditions (Figure 3d; Table 3). Marginal and significant positive cwmSLA–NDF relationships were observed under late‐season ambient and drought conditions, respectively (Figure 3e; Table 3).

**FIGURE 4:**
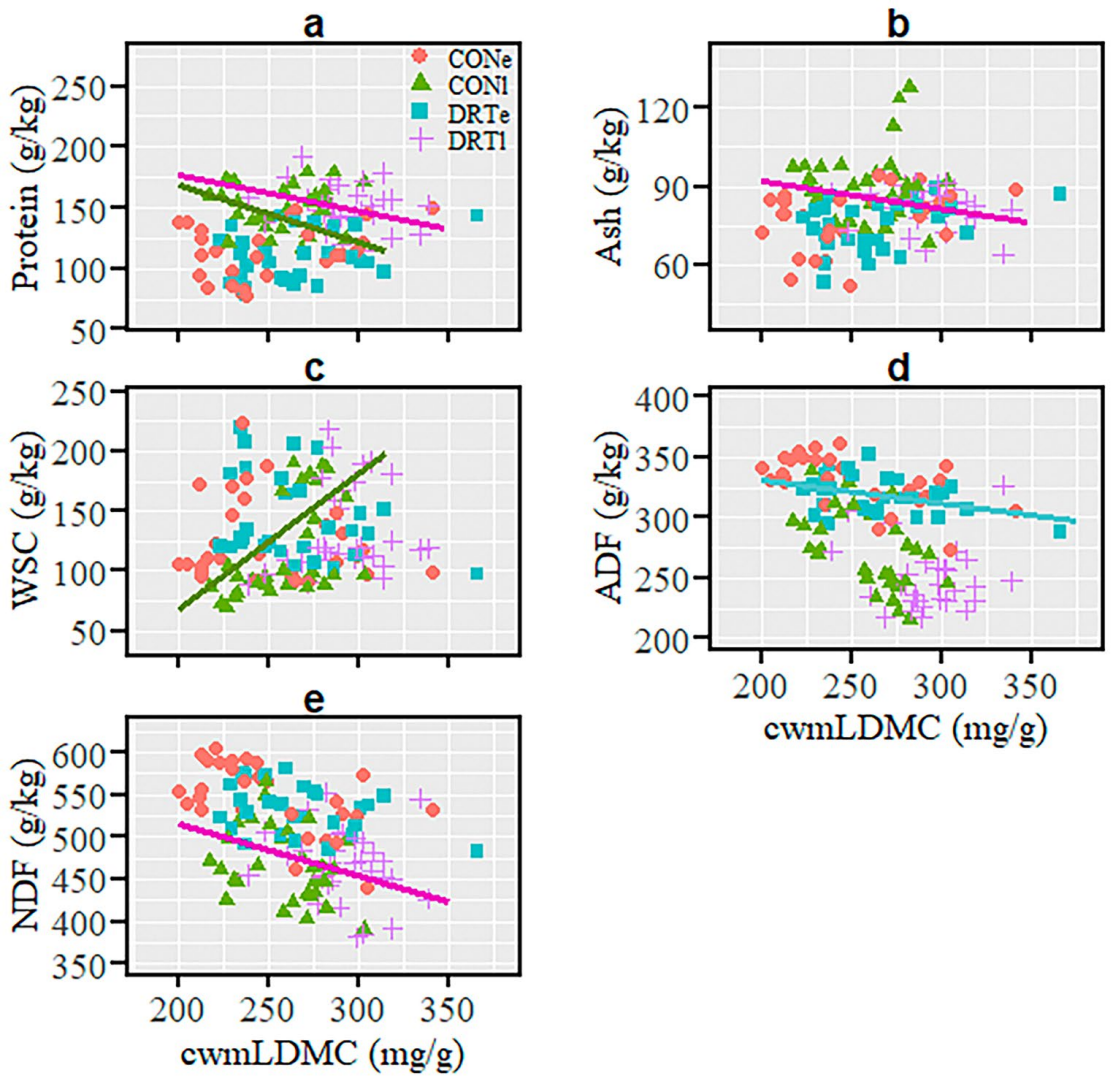
Relationships between community‐weighted mean leaf dry matter content (cwmLDMC) and forage quality parameters across three mountain grasslands at different times of the season (early and late) over 2 years. Only relationships (regression line) with significant (*p* < 0.05) or marginal (*p* < 0.1) *p*‐values are shown in the panels a–e while the full results are presented in Table 4. Each colour of the data points and regression lines represents each treatment, as indicated in the legends. CONe is ambient (control) conditions at early season period (i.e., before peak of biomass production); CONl is ambient (control) conditions at late season period (i.e., after peak of biomass production); DRTe is drought treatment conditions at early season period and DRTl is drought treatment conditions at late season period. ADF, acid detergent fibre; NDF, neutral detergent fibre; WSC, water soluble carbohydrate.

In early season, cwmLDMC had no relationship with protein, ash, WSC, and NDF contents under ambient and drought conditions (Figure 4a–c,e; Table 4). While the cwmLDMC–ADF relationship was not detected under early‐season ambient conditions, a marginally negative relationship was found under early drought (Figure 4d; Table 4). In late season, negative cwmLDMC–protein relationships were observed under both conditions (Figure 4a; Table 4). Ash content and cwmLDMC were unrelated under late‐season ambient conditions, but they were negatively related under late drought conditions (Figure 4b; Table 4). While a positive cwmLDMC–WSC relationship was observed under late‐season ambient conditions, such a relationship was not observed under late‐season drought conditions (Figure 4c; Table 4). While cwmLDMC had no relationship with ADF under both late‐season ambient and drought conditions (Figure 4d; Table 4), it had a marginally negative relationship with NDF under late‐season drought (Figure 4e; Table 4).

## 4 Discussion

### 4.1 Forage Quality and Leaf Traits Changed Over the Growing Season

Regarding our first hypothesis, we expected a general decrease in forage quality (i.e., decrease in internal cellular constituents and increase in cell wall components) from early to late season under ambient conditions, as widely reported (Lee et al. 2017; Jensen et al. 2017; Lemaire and Belanger 2020). This hypothesis was also based on the expectation that higher temperature and vapour pressure deficit (VPD) in late season (Table S1; Buttler et al. 2019) would promote increased lignification, reduced leaf nitrogen content, and senescence. Instead, the late‐season forage protein and ash contents were higher than those of early season, while soluble sugar (WSC) and both fibre contents were higher in early season across the sites. This same pattern was observed for all the assessed parameters, except WSC, within the three sites, despite that their species composition relatively differ. Soluble sugar did not change between the two periods at the driest/hottest site, decreased at the intermediate site, and increased at the wettest/coldest site. Regardless of the site‐specific sugar responses, there was a general decrease in forage fibres and increases in protein and ash contents in late season at all sites. Although our ash content determination method did not distinguish between nutritive and non‐nutritive minerals (Moles et al. 2011), the increased protein and decreased fibre contents in late season suggest an overall improvement in forage quality during the late season.

The higher fibre contents in early season contradict the common reports of increasing cell wall rigidity with plant age (Hamann 2012; Jensen et al. 2017; Ezquer et al. 2020). This may, in part, be due to the mowing management at the end of the early season period (Tasset et al. 2019). It is also possible that the post‐mowing plants' (re)growth towards the end of the season was slow compared to the beginning of the season. The favourable meteorological conditions (i.e., lower vapour pressure deficit), particularly in both the driest and intermediate sites (Table S1), likely accelerated most plants' phenological maturity during the early season compared to late‐season (re)growth (Buttler et al. 2019). The better growth conditions in the early season at the driest and intermediate sites possibly enabled grasses to grow faster and outcompete legumes and non‐legume forbs. As such, grasses, which are often more fibrous and less protein‐rich than legumes and forbs, disproportionately formed the bulk of the herbage in early season (Table S2; Sternberg et al. 1999). However, in the warmer and drier conditions of late season, slower growth of grasses reduced their competitiveness and enhanced the proportion of legumes and forbs in the forage yield (Table S2; Skinner et al. 2004). Hence, the studied grasslands are characterised by higher forage yield and lower quality in early season but lower yield (Meisser et al. 2019; Vitra et al. 2019) and higher quality in late season.

Consistent with our first hypothesis, our results revealed that the community‐weighted specific leaf area (cwmSLA) decreased from early to late season under ambient conditions across and within the least and medium wet sites (with similar species composition) but did not change at the wettest site that had distinct species composition. The leaf area of the dominant or all species possibly increased from the onset of the growing season until the peak of biomass production but then decreased, or remained stable, thereafter (Table S3) to maintain high water balance as vapor pressure deficit and temperature increased towards the end of the growing season (Liu et al. 2017; Wellstein et al. 2017; Firn et al. 2019; Gong and Gao 2019; Wang et al. 2022). This aligns with the climatic conditions of the study sites, where ambient precipitation was not limiting during the early season due to lower air temperatures, but became limiting in the late season as higher temperatures increased vapor pressure deficit (Table S1) and plant water stress (Buttler et al. 2019). Unlike the leaf area, we anticipated higher community‐weighted leaf dry matter content (cwmLDMC) during the late season, as plants would invest heavily in cell wall components to withstand the late‐season high water stress (Jiang et al. 2017) due to high vapor pressure deficit. In line with our hypothesis, we found that cwmLDMC was higher in late season across sites and in the least and medium wet sites that had similar species composition. However, cwmLDMC did not change with season at the wettest site, which has distinct species composition. Regardless of the site level differences, high or stable cwmLDMC possibly supported low transpiration rate and high water conservation in plants (Poorter et al. 2009; Suter and Edwards 2013; Wellstein et al. 2017), which slowed growth and aided the greater forage quality under late‐season ambient conditions.

### 4.2 Early‐ or Late‐Season Drought Imposed Limited Negative Effects on Forage Quality

Under both drought conditions, we expected a decline in forage quality (i.e., a substantial decline in internal cellular constituents versus increase in cell wall components), as cwmSLA and cwmLDMC would decrease and increase, respectively (second hypothesis). As expected, leaf traits exhibited conservative strategies across sites under both droughts; however, both droughts unexpectedly had limited negative effects on forage quality. Specifically, only early drought impacted and slightly decreased protein; both droughts increased WSC, decreased ash, and ADF; and neither drought altered NDF. The pattern of early or late drought impacts on the forage quality parameters and leaf traits differs among the three sites, but neither drought had a large negative effect on forage quality (i.e., no large change in internal cellular characteristics) in any site. Like our results, a meta‐analysis has shown that drought may have little or no negative effect on forage quality (Dumont et al. 2015).

Our cross and within sites results suggest that the plants, to an extent, maintained their internal cellular contents (i.e., unchanged or slightly decreased protein, increased WSC) at the expense of the cell wall components (i.e., decreased and stable ADF and NDF, respectively) under drought (Buxton 1996; Deléglise et al. 2015; Catunda et al. 2022). This possibly occurred via the efficient water conservation (i.e., stable or decreased cwmSLA, and stable or increased cwmLDMC) exhibited by the plants under drought conditions (Nord and Lynch 2009; Wellstein et al. 2017). The increase in WSC, which is a protective compound that mediates water stress, under both early and late season droughts also implies that most plants prioritised their survival over resource allocation for optimal growth under droughts (Volaire 1995; Sanada et al. 2007; Keep et al. 2021; Signori‐Müller et al. 2021). Moreover, the increase in WSC indicates an increase in digestible carbohydrate and readily available energy for herbivores utilizing the forages in such grasslands (Lee et al. 2002). Overall, the lack of substantial shift in the botanical composition (Table S2) in our drought plots may have contributed to the little change in community forage quality (Dumont et al. 2015).

### 4.3 Leaf Traits Predicted Forage Quality Under Certain Conditions

Under early‐ or late‐season ambient conditions, we anticipated communities with higher cwmSLA and lower cwmLDMC to have higher forage quality (i.e., higher internal cell contents and lower cell wall contents) than communities with lower cwmSLA and higher cwmLDMC (third hypothesis). Instead, the two leaf traits did not explain the differences in forage quality among different grassland communities under early‐season ambient conditions. Under late‐season ambient conditions, cwmSLA was positively associated with both fibre variables, while cwmLDMC negatively and positively related to protein and WSC, respectively. These detected relationships indicate that the communities dominated by higher leaf area plants possessed higher fibre forage and those dominated by higher LDMC plants provided forage of lower protein and higher sugar contents under the late‐season ambient conditions. These relationships jointly suggest that lower cwmSLA and cwmLDMC indicate better forage quality (i.e., relatively less fibre contents and relatively high protein content) in the studied grasslands under late‐season ambient conditions (i.e., when cwmLDMC was higher in the growing season; see Figure 2). While this finding agrees with previous reports that lower cwmLDMC may indicate higher forage quality (Gardarin et al. 2014; Tasset et al. 2019), it also provides new evidence that higher cwmSLA may indicate lower forage quality. Thus, the patterns of relationships between community‐weighted leaf traits and community forage quality may depend on the species or functional composition (Gardarin et al. 2014; Tasset et al. 2019) and environmental context (Al Haj Khaled et al. 2006).

Unlike the lack of relationships under early‐season ambient conditions, we detected higher protein and lesser fibre contents in communities with lower cwmSLA relative to those with higher cwmSLA under early drought conditions. We also noted that the lower cwmLDMC communities had higher ADF content than those with higher cwmLDMC under early drought. Thus, we infer that the communities with lower cwmSLA and higher cwmLDMC had higher forage quality (i.e., higher protein and lesser non‐readily digestible fibre contents) under early‐season extreme drought. The communities with higher forage quality might be dominated by plants that exhibited maximum water conservation (Wright et al. 2004; Poorter et al. 2009; Suter and Edwards 2013; Wellstein et al. 2017) and slower build‐up of non‐readily digestible fibre contents under early‐season drought. Although we did not assess the forage quality of each species or functional group, we also speculate that the observed relationships were, at least in part, driven by the proportion of legumes, forbs, and grasses in herbage harvested in different communities (Bruinenberg et al. 2002; Dindová et al. 2019). This is possible if the different plant functional groups in the grassland communities exhibited different adaptations to drought (Wu et al. 2023; Yan et al. 2019). As such, the lower leaf area communities possibly had a relatively high dominance and/or yield of protein‐rich legumes and forbs that enhanced the community‐level protein content (Dindová et al. 2019; Tasset et al. 2019). In contrast, the higher cwmLDMC communities might be constituted by a lower dominance or yield of high resource acquisitive or exploitative grasses that decreased the community‐level growth and fibres compared to the lower cwmLDMC communities (Bruinenberg et al. 2002; Wright et al. 2004; Dindová et al. 2019; Tasset et al. 2019).

Contrary to our third hypothesis but consistent with some relationships observed under late‐season ambient conditions, lower cwmSLA was associated with lower fibre contents (NDF and ADF) while lower cwmLDMC corresponded to higher protein, ash, and digestible NDF content under late drought conditions. These relationships collectively suggest that communities with lower cwmSLA and cwmLDMC had higher quality forage (i.e., higher protein and ash, more digestible fibre) than those with higher cwmSLA and cwmLDMC under late‐season drought conditions. The latter communities may have had lower forage quality because their plants thrived and exhibited fast, acquisitive growth under late‐season drought. To support the fast growth, the plants potentially increased their leaf area to optimise photosynthesis and allocate photosynthates to other organs (Chaves et al. 2002; Donovan et al. 2007; Nippert and Knapp 2007) while increasing LDMC to mitigate the high transpiration associated with higher SLA under late‐season drought (Suter and Edwards 2013; Volaire et al. 2014). In contrast, the communities with higher forage quality presumably experienced slower growth under late drought by reducing transpiration and photosynthesis through lower LDMC and SLA, respectively (Nord and Lynch 2009; Wellstein et al. 2017; Hartzell 2019; Daningsih et al. 2022).

## 5 Conclusions

Contrary to expectations, community forage quality was higher under late‐season ambient conditions than during the early season across the studied grasslands, with increased protein and ash contents and lower fibre content. This was likely due to the reduced growth associated with lower community‐weighted SLA and higher LDMC in the late season. Both early‐ and late‐season extreme droughts had limited negative effects on forage quality, as they did not greatly alter leaf traits or plants' water acquisition capacity (i.e., less decrease in cwmSLA or less increase in cwmLDMC), contradicting our expectations. In contrast to expectations, the leaf traits and forage quality parameters were unrelated under early‐season ambient conditions, while lower cwmSLA and higher cwmLDMC indicated higher community forage quality, particularly in terms of protein, ash, and less non‐readily digestible fibre contents, under early drought conditions. Under late‐season ambient or drought conditions, lower cwmSLA and cwmLDMC indicated higher forage quality in terms of higher protein and ash and more digestible fibre contents. Overall, our results suggest that the dynamics of leaf economic traits can predict forage quality patterns in grasslands under certain circumstances, including regular intra‐seasonal dry periods and extreme drought conditions. As studies linking leaf economic traits and forage quality under extreme drought scenarios remain scarce, further research is needed to improve our understanding of how climate change affects herbivore health and associated ecosystem functions.

## Author Contributions

**Taofeek O. Muraina:** formal analysis (lead), visualization (lead), writing – original draft (lead), writing – review and editing (lead). **Amarante Vitra:** conceptualization (supporting), data curation (lead), investigation (lead), methodology (equal), writing – review and editing (supporting). **Massimiliano Probo:** formal analysis (supporting), writing – review and editing (supporting). **Jason P. Martina:** formal analysis (supporting), writing – review and editing (supporting). **Alexandre Buttler:** conceptualization (lead), formal analysis (supporting), funding acquisition (lead), methodology (equal), supervision (lead), writing – review and editing (supporting). **Pierre Mariotte:** data curation (supporting), formal analysis (supporting), investigation (equal), supervision (supporting), writing – review and editing (equal).

## Conflicts of Interest

The authors declare no conflicts of interest.

## Supporting information


Data S1.

## Data Availability

Data is available in the Dryad Digital Repository https://doi.org/10.5061/dryad.3xsj3txtd.
