# Supplementary material for: Linking Leaf Economic Traits With Forage Quality Across Temperate Grasslands Under Ambient and Drought Conditions
Source: Ecol Evol. 2025 Jun 24;15(6):e71569. doi: 10.1002/ece3.71569 (PMC12186133; doi:10.1002/ece3.71569)
Supplement: Supplementary file 1 — Data S1. [file ECE3-15-e71569-s001.docx]

**Linking leaf economic traits with forage quality across temperate grasslands under ambient and drought conditions**

Taofeek O. Muraina^1,2^*, Amarante Vitra^3,4,5^, Massimiliano Probo^6^, Jason P. Martina^1^, Alexandre Buttler^3,4^, Pierre Mariotte^3,4,6^

^1^ Department of Biology, Texas State University, San Marcos, 78666, United States of America.

^2^ Department of Animal Health and Production, Oyo State College of Agriculture and Technology, P.M.B. 10, Igbo-Ora, Oyo State, Nigeria.

^3^ Ecole Polytechnique Fédérale de Lausanne (EPFL), School of Architecture, Civil and Environmental Engineering (ENAC), Laboratory of Ecological Systems (ECOS), Station 2, 1015 Lausanne, Switzerland.

^4^ Swiss Federal Institute for Forest, Snow and Landscape Research (WSL), Site Lausanne, Case postale 96, 1015 Lausanne, Switzerland.

^5^ Department of Livestock Sciences, Research Institute of Organic Agriculture (FiBL), Ackerstrasse 113, 5070 Frick, Switzerland

^6^ Agroscope, Grazing Systems Group, Route de la Tioleyre 4, CH-1725 Posieux, Switzerland.

*Corresponding author: [too.muraina@gmail.com](mailto:too.muraina@gmail.com)

**Table S1.** Dates of the year, mean daily air temperature and its deviation from the long-term average (MeteoSwiss, 1985-2014), as well as mean vapour pressure deficit (VPD) at the three study sites during the early- and late-season growth periods of the two experimental years (2015 and 2016).

| **Year** | **Site** | **Time** | **Dates**  **(day.month)** | **Air temp. (°C)** | **Norm deviation**  **(°C)** | **VPD (Kpa)** |
| --- | --- | --- | --- | --- | --- | --- |
| 2015 | Site A | Early | 31.03 to 31.05 | 13.9 | + 2.3 | 0.71 |
|  |  | Late | 01.06 to 31.07 | 21.2 | + 2.8 | 1.28 |
|  | Site B | Early | 23.04 to 22.06 | 13 | - 1.5 | 0.48 |
|  |  | Late | 23.06 to 22.08 | 19.2 | + 2.7 | 0.95 |
|  | Site C | Early | 27.05 to 25.07 | 11.4 | - 0.8 | 0.49 |
|  |  | Late | 26.07 to 25.09 | 14.4 | + 1.5 | 0.28 |
| 2016 | Site A | Early | 09.04 to 08.06 | 10.5 | + 1.9 | 0.48 |
|  |  | Late | 09.06 to 08.08 | 17.9 | - 1.0 | 1.78 |
|  | Site B | Early | 21.04 to 20.06 | 9.9 | - 1.6 | 0.27 |
|  |  | Late | 21.06 to 20.08 | 16.7 | + 0.3 | 0.59 |
|  | Site C | Early | 13.05 to 12.07 | 10.4 | - 0.4 | 0.19 |
|  |  | Late | 13.07 to 12.09 | 13.5 | - 0.1 | 0.33 |

Site A: Chéserex, Site B: Saint-George, and Site C: Trois Chalets. Early season- before peak of plant biomass production; Late season- after peak of plant biomass production.

**Table S2.** Average percentage species composition at the three study sites under early- and late-season ambient and drought conditions over two years (2015 and 2016).

| **Site** | **Plant group** | **Early season** | | **Late season** | |
| --- | --- | --- | --- | --- | --- |
|  |  | **Control** | **Drought** | **Control** | **Drought** |
| **Site A** | Dominant | 66.19% | 65.58% | 69.04% | 74.14% |
|  | Non-dominant | 33.81% | 34.42% | 30.96% | 25.86% |
|  | Grass | 77.22% | 73.95% | 61.85% | 65.38% |
|  | Forbs | 12.71% | 13.19% | 25.55% | 18.03% |
|  | Legumes | 10.07% | 12.85% | 12.61% | 16.59% |
| **Site B** | Dominant | 65.40% | 63.85% | 76.66% | 85.40% |
|  | Non-dominant | 34.60% | 36.15% | 23.34% | 14.60% |
|  | Grass | 71.29% | 69.38% | 61.22% | 67.34% |
|  | Forbs | 8.03% | 9.45% | 12.53% | 7.95% |
|  | Legumes | 20.68% | 21.17% | 26.25% | 24.72% |
| **Site C** | Dominant | 77.13% | 80.96% | 85.33% | 85.17% |
|  | Non-dominant | 22.87% | 19.04% | 14.67% | 14.83% |
|  |  |  |  |  |  |
|  | Grass | 51.83% | 54.73% | 65.62% | 65.58% |
|  | Forbs | 39.89% | 37.31% | 27.32% | 25.55% |
|  | Legumes | 8.28% | 7.96% | 7.06% | 8.87% |

**Table S3.** Traits’ values of dominant species at the three study sites under early- and late-season ambient and drought conditions over two years (2015 and 2016).

|  |  |  | **EARLY SEASON** | | | | **LATE SEASON** | | | |
| --- | --- | --- | --- | --- | --- | --- | --- | --- | --- | --- |
|  |  |  | **SLA (cm^2^/g)** | | **LDMC (mg/g)** | | **SLA (cm^2^/g)** | | **LDMC (mg/g)** | |
| **Site** | **Year** | **Dominant species** | **Control** | **Drought** | **Control** | **Drought** | **Control** | **Drought** | **Control** | **Drought** |
| **Site A** | **2015** | *D. glomerata* | 29.50 | 28.17 | 297.72 | 288.44 | 18.69 | 17.10 | 321.36 | 341.81 |
|  |  | *L. perenne* | 29.32 | 26.14 | 224.07 | 235.97 | 19.00 | 15.30 | 245.49 | 278.05 |
|  |  | *P. pratense* | 22.72 | 23.44 | 307.83 | 307.21 | 21.34 | 19.39 | 319.82 | 341.85 |
|  |  | *P. pratensis* | 42.02 | 42.89 | 206.60 | 261.40 | 18.72 | 17.22 | 341.99 | 369.90 |
|  |  | *T. officinale* | 37.42 | 32.75 | 158.36 | 172.55 | 23.73 | 19.94 | 227.01 | 259.41 |
|  |  | *T. repens* | 33.69 | 31.62 | 202.01 | 221.03 | 23.17 | 19.89 | 242.80 | 267.40 |
|  | **2016** | *D. glomerata* | 26.43 | 25.88 | 310.15 | 288.75 | 21.11 | 20.50 | 279.73 | 294.73 |
|  |  | *L. perenne* | 29.72 | 24.77 | 231.00 | 227.83 | 21.80 | 21.23 | 269.37 | 270.56 |
|  |  | *P. pratense* | 25.44 | 22.71 | 294.97 | 308.29 | 21.45 | 22.24 | 343.98 | 333.57 |
|  |  | *P. pratensis* | 50.09 | 47.28 | 240.94 | 262.76 | 22.17 | 20.55 | 303.62 | 349.65 |
|  |  | *T. officinale* | 36.64 | 31.61 | 134.72 | 156.13 | 29.72 | 25.51 | 159.92 | 187.18 |
|  |  | *T. repens* | 41.15 | 34.76 | 186.91 | 196.36 | 32.14 | 26.96 | 240.76 | 229.11 |
| **Site B** | **2015** | *D. glomerata* | 35.50 | 34.49 | 274.74 | 294.61 | 24.79 | 19.58 | 267.37 | 328.44 |
|  |  | *L. perenne* | 32.77 | 28.03 | 209.43 | 250.22 | 24.14 | 17.70 | 194.03 | 250.70 |
|  |  | *P. pratensis* | 43.00 | 42.22 | 277.73 | 267.27 | 23.44 | - | 287.96 | - |
|  |  | *T. repens* | 34.00 | 30.80 | 198.72 | 215.94 | 27.85 | 18.69 | 208.41 | 272.44 |
|  | **2016** | *D. glomerata* | 27.97 | 30.25 | 296.29 | 324.24 | 23.21 | 20.99 | 263.09 | 324.14 |
|  |  | *L. perenne* | 29.72 | 28.45 | 193.25 | 235.60 | 25.20 | 19.83 | 250.86 | 293.41 |
|  |  | *P. pratensis* | 38.62 | 35.20 | 256.12 | 287.89 | 23.45 | 18.10 | 305.29 | 362.55 |
|  |  | *T. repens* | 36.94 | 33.69 | 175.50 | 194.81 | 28.87 | 23.52 | 217.51 | 245.14 |
| **Site C** | **2015** | *A. capillaris* | 27.81 | 27.86 | 295.36 | 299.06 | 27.91 | 26.41 | 294.97 | 305.83 |
|  |  | *A. vulgaris* | 20.62 | 20.09 | 294.79 | 313.41 | 19.49 | 19.34 | 281.15 | 289.16 |
|  |  | *F. rubra* | 15.61 | 15.63 | 332.02 | 331.37 | 17.92 | 16.22 | 287.46 | 308.73 |
|  |  | *R. acris* | 25.03 | 21.26 | 221.85 | 251.63 | 22.03 | 20.79 | 214.95 | 231.07 |
|  |  | *T. repens* | 29.49 | 27.80 | 233.16 | 238.16 | 26.18 | 28.24 | 229.03 | 224.85 |
|  | **2016** | *A. capillaris* | 26.96 | 25.41 | 319.50 | 338.99 | 27.59 | 22.89 | 288.45 | 332.13 |
|  |  | *A. vulgaris* | 21.81 | 20.80 | 325.61 | 283.36 | 20.42 | 18.72 | 283.44 | 310.71 |
|  |  | *F. rubra* | 21.66 | 20.27 | 287.89 | 275.26 | 14.37 | 11.46 | 303.06 | 343.69 |
|  |  | *R. acris* | 25.56 | 23.74 | 221.04 | 228.24 | 22.01 | 18.97 | 222.90 | 250.46 |
|  |  | *T. repens* | 34.10 | 33.93 | 230.90 | 222.43 | 25.04 | 23.37 | 258.60 | 246.13 |

Site A: Chéserex, Site B: Saint-George, and Site C: Trois Chalets. **Dominant species names**: *Dactylis glomerata; Lolium perenne; Phleum pratense; Poa pratensis; Taraxacum officinale; Trifolium repens; Agrostis capillaris; Alchemilla vulgaris; Festuca rubra; Ranunculus acris.* **Note:** In late season of 2015 at site B, leaves of *Poa pratensis* were not collected under drought because the leaves were too dry.


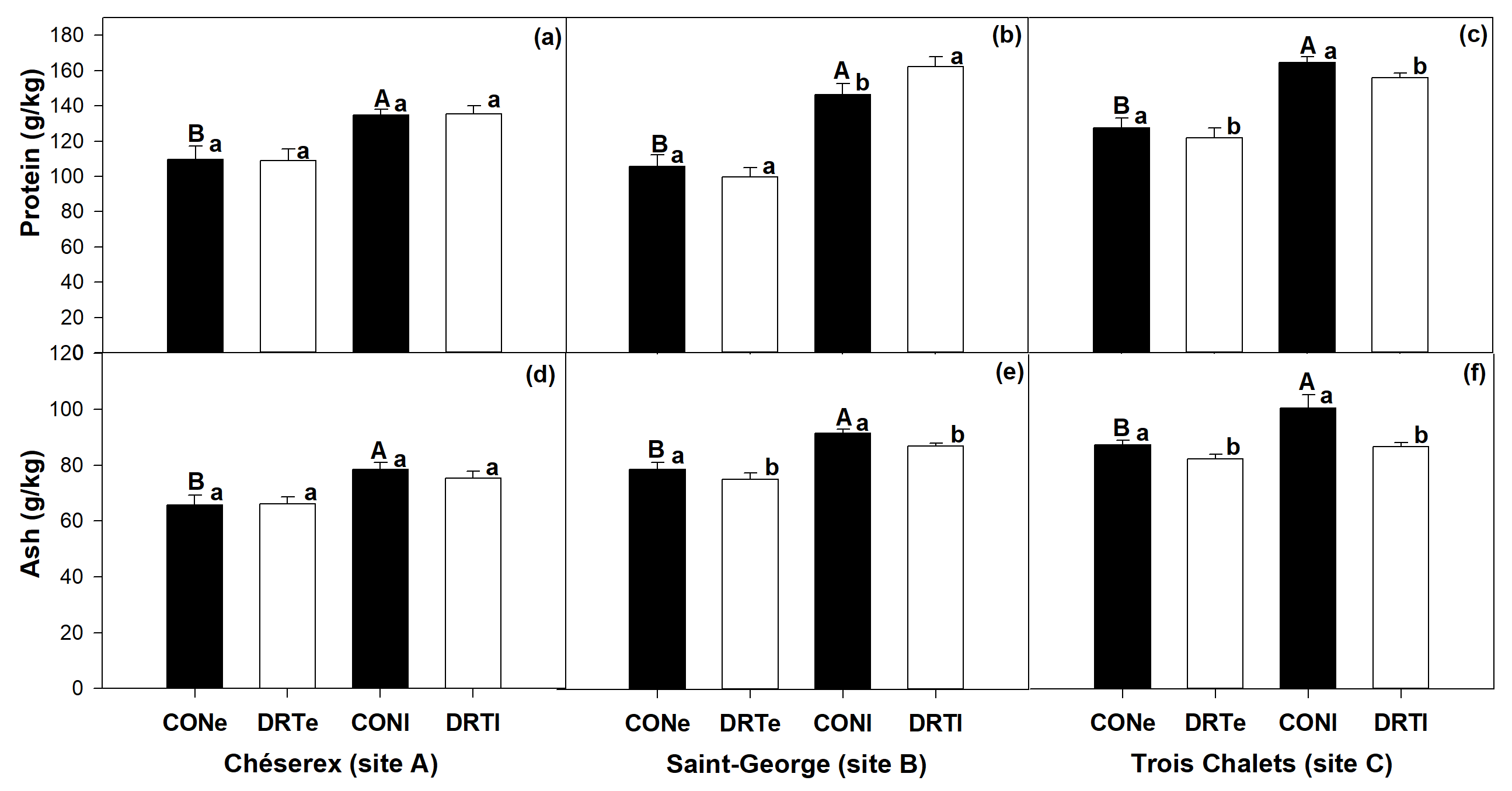
**Fig. S1:** Drought effects on forage protein and ash contents within three mountain grasslands in early and late season periods. Average values over the years 2015 and 2016 and SE are given. CONe is ambient (control) conditions at early season period (i.e., before peak of biomass production); CONl is ambient (control) conditions at late season period (i.e., after peak of biomass production); DRTe is drought treatment conditions at early season period and DRTl is drought treatment conditions at late season period. Different upper-case letters on two black bars in a panel (site) indicate significant (P < 0.05) or marginal (P < 0.1) differences between early and late season periods under ambient conditions (i.e., control plots) (Table 1). Different lower-case letters on black vs. white bars in a panel (site) indicate significant or marginal differences between control and drought treatments at either early or late period of the season (Table 2).


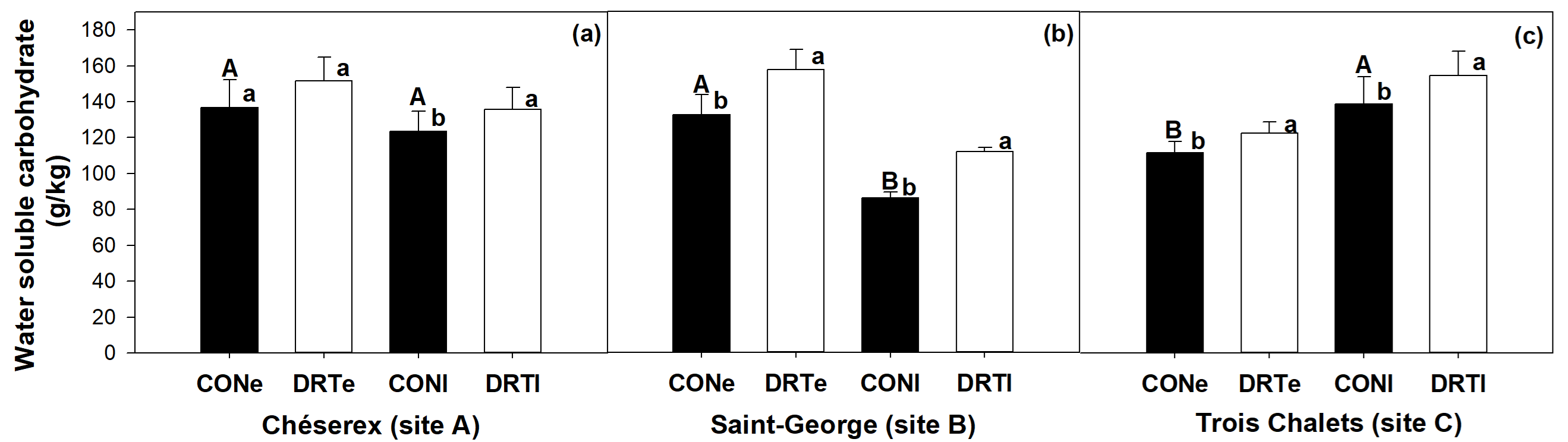
**Fig. S2:** Drought effects on forage water soluble carbohydrate within three mountain grasslands in early and late season periods of the season. Average values over the years 2015 and 2016 and SE are given. CONe is ambient (control) conditions at early season period (i.e., before peak of biomass production); CONl is ambient (control) conditions at late season period (i.e., after peak of biomass production); DRTe is drought treatment conditions at early season period and DRTl is drought treatment conditions at late season period. Different upper-case letters on two black bars in a panel (or site) indicate significant (P < 0.05) or marginal (P < 0.1) differences between early and late season periods under ambient conditions (i.e., control plots) (Table 1). Different lower-case letters on black vs. white bar in a panel (or site) indicate significant or marginal differences between control and drought treatments at either early or late period of the season (Table 2).


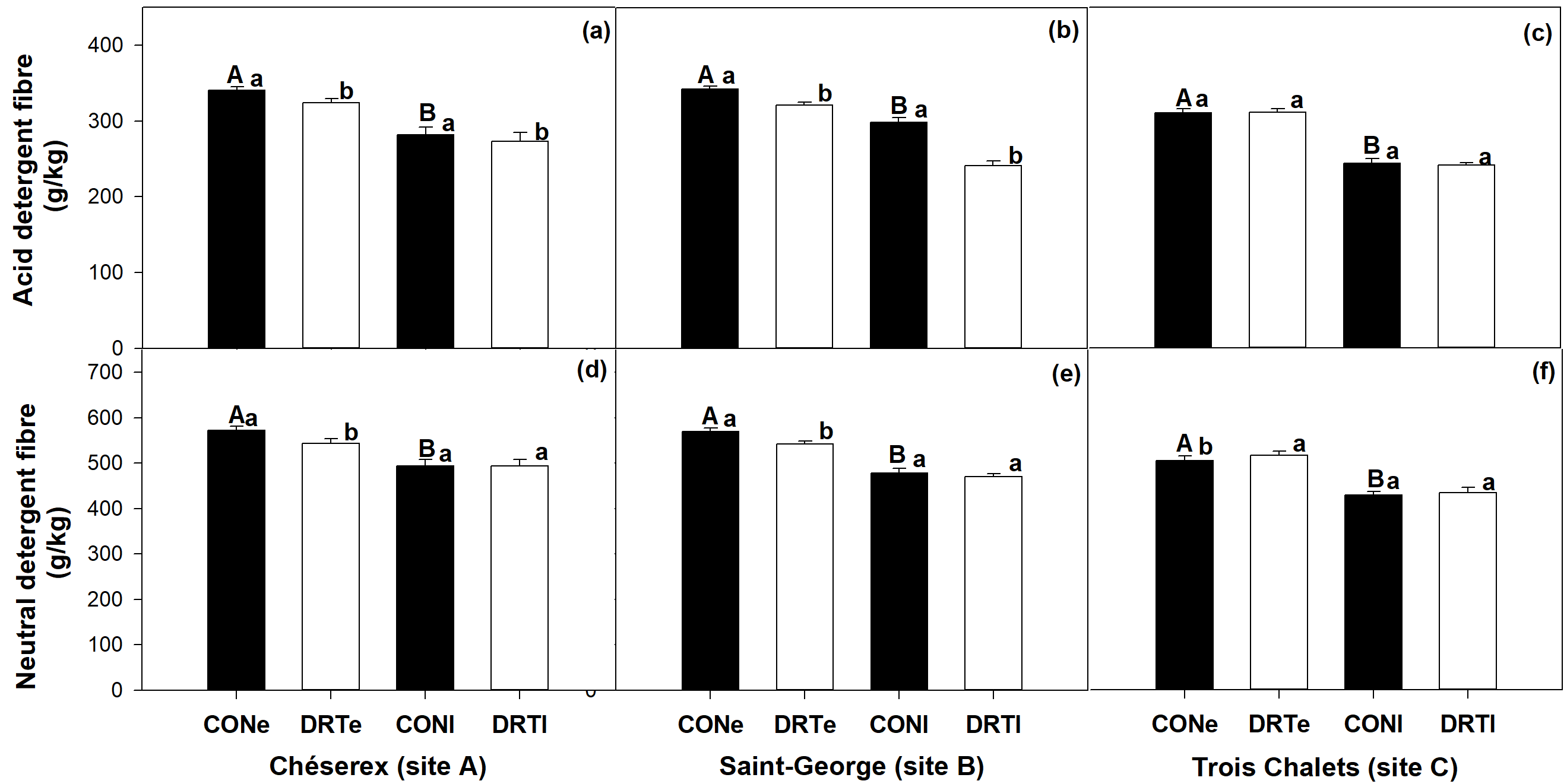
**Fig. S3:** Drought effects on forage fibre contents within three mountain grasslands in early and late season periods of the season. Average values over the years 2015 and 2016 and SE are given. CONe is ambient (control) conditions at early season period (i.e., before peak of biomass production); CONl is ambient (control) conditions at late season period (i.e., after peak biomass of production); DRTe is drought treatment conditions at early season period and DRTl is drought treatment conditions at late season period. Different upper-case letters on two black bars in a panel (or site) indicate significant (P < 0.05) or marginal (P < 0.1) differences between early and late season periods under ambient conditions (i.e., control plots) (Table 1). Different lower-case letters on black vs. white bar in a panel (or site) indicate significant differences between control and drought treatments at either early or late period of the season (Table 2).


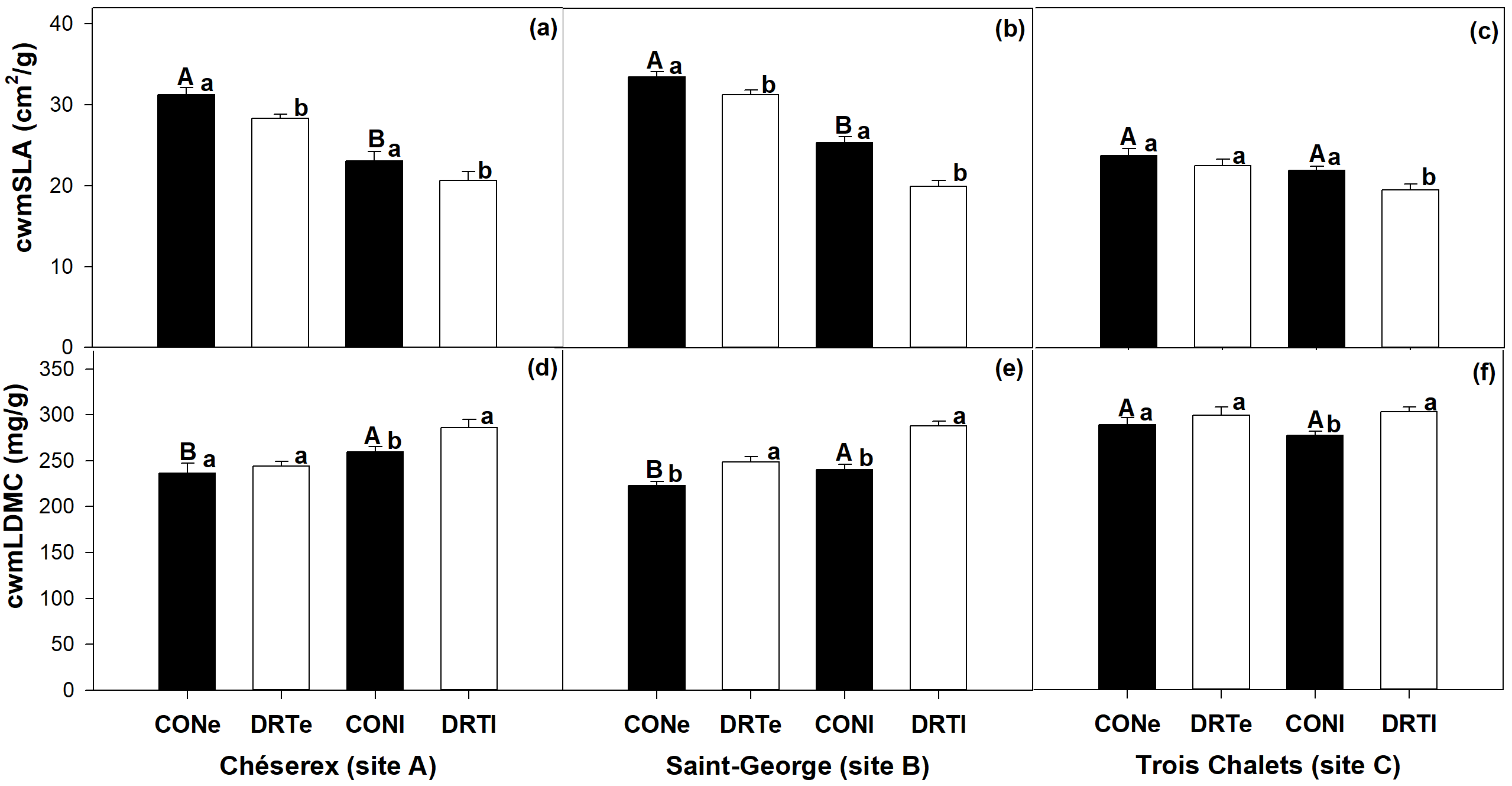
**Fig. S4:** Drought effects on community-weighted leaf traits within three mountain grasslands in early and late season periods of the season. Average values over the years 2015 and 2016 and SE are given. CONe is ambient (control) conditions at early season period (i.e., before peak of biomass production); CONl is ambient (control) conditions at late season period (i.e., after peak of biomass production); DRTe is drought treatment conditions at early season period and DRTl is drought treatment conditions at late season period. Different upper-case letters on two black bars in a panel (site) indicate significant (P < 0.05) or marginal (P < 0.1) differences between early and late season periods under ambient conditions (i.e., control plots) (Table 1). Different lower-case letters on black vs. white bar in a panel (site) indicate significant differences between control and drought treatments at either early or late period of the season. (Table 2).
